# Black, Asian and minority ethnic women's experiences of maternity services in the UK: A qualitative evidence synthesis

**DOI:** 10.1111/jan.15233

**Published:** 2022-03-24

**Authors:** Jennifer MacLellan, Sarah Collins, Margaret Myatt, Catherine Pope, Wanja Knighton, Tanvi Rai

**Affiliations:** ^1^ Nuffield Department of Primary Care Health Sciences University of Oxford, Radcliffe Observatory Quarter Oxford UK; ^2^ Nuffield Department of Women's & Reproductive Health University of Oxford, Level 3, Women's Centre, John Radcliffe Hospital Oxford UK; ^3^ University Hospitals Sussex NHS Foundation Trust Worthing UK; ^4^ Riseproject.co. Oxford UK

**Keywords:** ethnic minority, literature review, maternity, meta‐synthesis, UK

## Abstract

**Aims:**

Black, Asian and minority ethnic women are at higher risk of dying during pregnancy, childbirth and postnatally and of experiencing premature birth, stillbirth or neonatal death compared with their White counterparts. Discrimination against women from ethnic minorities is known to negatively impact women's ability to speak up, be heard and their experiences of care. This evidence synthesis analysed Black, Asian and minority ethnic women's experiences of UK maternity services in light of these outcomes.

**Design:**

We conducted a systematic review and qualitative evidence synthesis using the method of Thomas and Harden.

**Data Sources:**

A comprehensive search in AMED, Cinahl, Embase, Medline, PubMed and PsycINFO, alongside research reports from UK maternity charities, was undertaken from 2000 until May 2021. Eligible studies included qualitative research about antenatal, intrapartum and postnatal care, with ethnic minority women in maternity settings of the UK NHS.

**Review Methods:**

Study quality was graded using the Critical Appraisal Skills Programme tool.

**Results:**

Twenty‐four studies met the inclusion criteria. Our synthesis highlights how discriminatory practices and communication failures in UK NHS maternity services are failing ethnic minority women.

**Conclusion:**

This synthesis finds evidence of mistreatment and poor care for ethnic minority women in the UK maternity system that may contribute to the poor outcomes reported by MBRRACE. Woman‐centred midwifery care is reported as positive for all women but is often experienced as an exception by ethnic minority women in the technocratic birthing system.

**Impact:**

Ethnic minority women report positive experiences when in receipt of woman‐centred midwifery care. Woman‐centred midwifery care is often the exception in the overstretched technocratic UK birthing system. Mistreatment and poor care reported by many ethnic minority women in the UK could inform the inequalities of outcomes identified in the MBRRACE report.

## 1 INTRODUCTION

The ‘Mothers and Babies: Reducing Risk through Audits and Confidential Enquiries’ (MBRRACE‐UK) audit confirmed that Black, Asian and minority ethnic women are at higher risk of dying during pregnancy, childbirth and postnatally and of experiencing premature birth, stillbirth or neonatal death compared with their White counterparts (Knight et al., 2020). Published explanations for these inequalities are complex and include a combination of contributing factors: organizational, language and cultural, help‐seeking and access barriers (Aquino et al., 2015, Fisher & Fraser, 2020, Henderson et al., 2018, Murray et al., 2010). Much of the evidence comes from international literature rather than the UK specifically and reflects different social, cultural and historical experiences. MBRRACE highlighted the need to review the evidence related to the UK health system.

## 2 BACKGROUND

Feeling cared for (Beake et al., 2013; Redshaw & Heikkila, 2011), staff attitudes (Rayment‐Jones et al., 2019) and communication (Harper Bulman & McCourt, 2002; Wikberg et al., 2012) have been reported as being important by the whole population of birthing women. Yet experiences of negative stereotyping and lack of ‘cultural competence’ among maternity staff reveal dimensions of poor care experiences unique to women from ethnic minorities (Jomeen & Redshaw, 2013). Stereotyping and pre‐conceived ideas about women from ethnic minorities negatively impact women's ability to speak up and their experience of care (Hoffman et al., 2016; Puttusery et al., 2008). Such attitudes reinforce a ‘Them and Us’ approach that expects ethnic minority women to adapt to an insensitive and sometimes discriminatory health system rather than the system being responsive to the needs of the women (Lyons et al., 2008). There is a significant body of literature (largely from the USA) that co‐implicates social, economical and political forces in producing the stigma and inequality experienced in negative encounters with health services. These negative encounters include attempts at service engagement being rebuffed (Davis, 2019; Metzl & Hansen, 2014; O'Mahony & Donnelly, 2010).

## 3 THE REVIEW

### 3.1 Aim

The aim of this review was to synthesize the published qualitative evidence about Black, Asian and minority ethnic women's experiences of UK maternity services in light of the disparities reported in maternity outcomes between different groups of women.

### 3.2 Design

We used a thematic evidence synthesis method to extend the interpretations offered in the original individual studies included in this review (Thomas & Harden, 2008).

### 3.3 Search method

**FIGURE 1:**
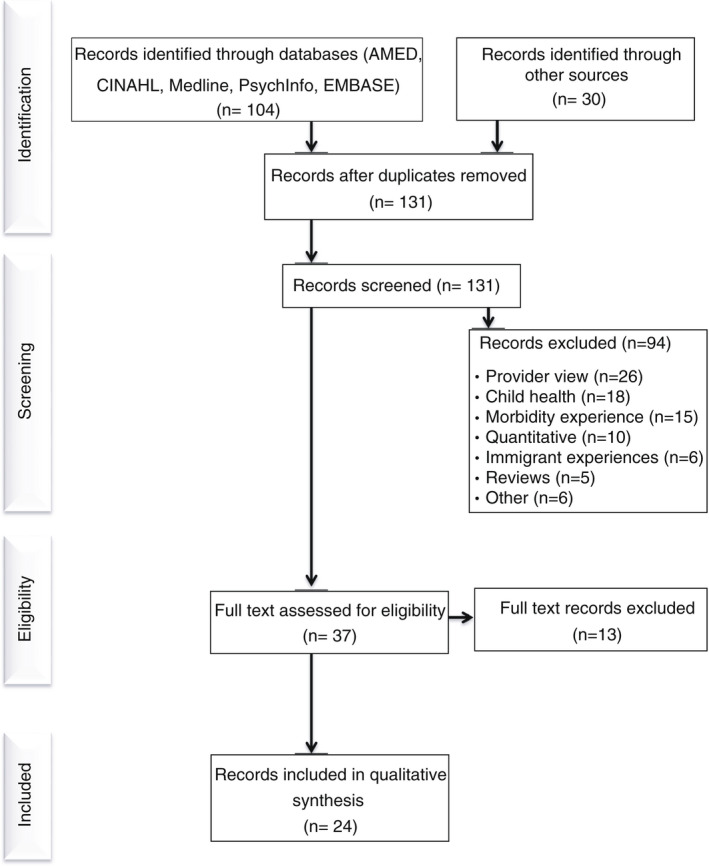
Literature search results

We registered our review on the PROSPERO database (MacLellan et al., 2020), followed the PRISMA guidance (Page et al., 2021; Rethlefsen et al., 2021) and used PICO (Population, Interest, COntext) to structure systematic searches for qualitative studies where Population = Black, Asian and minority ethnic women, Interest = Experiences, COntext = UK maternity services (Miller, 2001). The acronym ‘BAME’ was used in searching due to its consistent use in the preceding 20 years, but in line with recent UK government guidance, we use the term ‘ethnic minorities’ to include Black, Asian and other minority ethnic people. This update in terminology is an acknowledgement that the concept of ‘race’ refers to a shared culture and history among a group of people rather than skin colour. While MBRRACE did not include this group in their description of racial inequalities, the inclusion of the experiences of white minoritized ethnicities such as Orthodox Jewish, Gypsy, Roma and Irish Travellers is appropriate to our synthesis (Race Disparity Unit, 2021). Searches were carried out in December 2020 in AMED, EMBASE, PsycINFO, CINAHL and MEDLINE databases, published in English from 2000. This cut‐off date was chosen as there was a major change in the maternity system approach in the late 1990s with the Changing Childbirth report, to woman‐centred care (Department of Health, 1993, 1997). For full search strategy, see Table 1: Search strategy. In addition, we used backwards citation tracking, Pubmed ‘related articles’, Google Scholar and research outputs of UK maternity and advocacy charities. Searches were repeated in May 2021 and yielded no additional records (Figure 1).

**TABLE 1:** Literature search terms

Population		Interest	Context
BAME or Black or Asian or Minority Ethnic	Maternity Service or Antenatal or Postnatal or Childbirth	Experience or Perception or Attitude or View	NHS or National Health Service or the UK or the United Kingdom
Orthodox Jewish, Jewish and Judaism			
Traveller community or travelling community or travellers or Gypsy traveller or Roma			

### 3.4 Search outcome

We included studies that reported qualitative (interpretive and textual) data about antenatal, intrapartum and postnatal care, with ethnic minority women in primary and secondary care settings. We excluded papers about the experience of asylum seekers and women without recourse to public funds due to their unique financial and immigration concerns. Other exclusions were papers focused on morbidity, child health or the impact of COVID‐19. Studies reporting only professional perspectives were also excluded.

### 3.5 Quality appraisal

Study quality was graded using the Critical Appraisal Skills Programme (CASP) tool for qualitative studies (CASP, 2019). Two reviewers (JM and SC) graded the papers independently, conferred on a random selection of 12 papers, with the third reviewer available to resolve any disparities (TR). While CASP does not advocate a scoring system, the majority of included papers achieved ‘yes’ on eight domains or more. As recommended by Atkins et al. (2008), papers were not excluded as a result of a low score but were integrated into the synthesis with these concerns made explicit. In all but one instance, the findings of the lower quality papers were corroborated by two or more high‐quality papers, adding confidence to the findings. In the single incidence, a confidence statement follows the report of the finding. We also examined the relative contributions of each study to the final analytic themes (Table 3: Theme contribution of included papers).

### 3.6 Data abstraction

We collated records into an Excel database, removed duplicates and screened abstracts for inclusion against the inclusion/exclusion criteria (JM and SC). Full texts were screened independently by two researchers (JM and SC) with a third reviewer available to resolve any discrepancies (TR).

### 3.7 Synthesis

Findings and results sections of papers were extracted and entered verbatim into NVivo 12 software to support thematic analysis guided by Thomas and Harden (2008). This began with independent line‐by‐line inductive coding by two researchers (JM and SC), who then organized the codes into broad descriptive themes for discussion by the full research team to refine. Four major themes have identified that structure the presentation of our synthesis findings below.

## 4 RESULTS

Searching yielded 131 papers after removing duplicates, with 37 fulfilling our eligibility criteria (Figure 1). Thirteen of these did not report directly on maternity service experiences, leaving 24 for synthesis (Table 2). Study samples ranged from 7 to 219 participants, with a combined total of over 760 participants from a range of different self‐identified ethnic backgrounds or classified according to researcher ethnic criteria (*n* = 2). The principal method of data collection was semi‐structured interview (*n* = 21) or focus groups (*n* = 5). An interpreter or a bi‐lingual researcher was offered in 18 studies, with the remaining six being conducted exclusively in English. Studies that scored high on researcher reflexivity used non‐hierarchical language, collected data through peer researchers or culturally congruent researchers, recruited participants through community sources and scored higher in terms of quality. Poorer quality studies tended to be more descriptive than interpretive but nonetheless offered some valuable data. The synthesis identified four core themes about the experience of maternity services in the UK by women of Black, Asian and minority ethnicities. These are

- birthing in a technocratic system
- communication failures
- mistreatment of women
- woman‐centred care as exceptional, not routine

**TABLE 2:** Characteristics of included studies

No.	References	No. of participants	Time since gave birth	Sample	Data collection method	Research methodology	Sampling	Reflexivity	CASP
1	Ali N. (2004) Experiences of Maternity Services: Muslim Women's Perspectives. London;The Maternity Alliance; [https://www.maternityaction.org.uk/wp‐]	43	In the last 3 years	Muslim mothers Bangladesh, Pakistan, Europe, Iraq, Somali, African, Indian	Focus group in chosen language with interpreter always present, creche provided	Mixed methods	Snowball through Muslim organizations and charities	None	8
2	Alshawish E, Marsden j, Yeowell G, Wibberley C. (2013) Investigating access to and use of maternity health‐care services in the UK by Palestinian women. British Journal of Midwifery 2013;21:8:571–577	22	Unknown	Palestinian women in the UK	Semist interview in English or Arabic	Pragmatism, framework analysis	Snowball at school and mosque	Yes	8
3	Beake S, Acosta L, Cooke P, McCourt C. (2013) Caseload midwifery in a multi‐ethnic community: The woman's experiences. Midwifery;29:996–1002	16	In the last 6 months	16 ethnic minority women: Black, Indian, Oriental, Bangladeshi, Other	Semi‐structured interview, only English speakers responded	Framework analysis	Invite letters sent in English	None	8
4	Binder‐Finnema P, Borne Y, Johnsdotter S, Essen B. (2012) Shared language is essential: Communication in a multiethnicity obstetric care setting. Journal of Health Communication 17:1171–1186	60	2 days–10 years ago	39 immigrant Somali, 11 Ghanaian, 10 White British women and 62 obstetric care providers	23 interviews, 5 focus group discussions using cultural brokers and interpreters	Framework analysis, naturalistic inquiry	Snowball in Somali through cultural brokers	None	7
5	Birthrights. (2020) Holding it all together: Understanding how far the human rights of women facing disadvantage are respected during pregnancy, birth, postnatal care. London: Birthrights	12	In the last 3 years	11 Black, Asian, minority ethnic women, 1 white European	Semi‐structured interviews with interpreter used in 1	Thematic analysis	Through targetted support services	Yes	9
6	Cardwell, V. Wainwright, L. (2019) Making Better Births a reality for women with multiple disadvantages. A qualitative peer research study exploring perinatal women's experiences of care and services in north‐east London. Birth Companions and Revolving Doors Agency London.	34	In the last 5 years	Women of multiple disadvantages including ethnic minority status	Peer‐led semi‐structured interviews and 1 focus group discussion. Interpreters provided	Participatory research	Through targetted support services	Use of peer researchers	10
7	Cross‐Sudworth, F. Williams, A. Herron‐Marx, S. (2011) Maternity services in multi‐cultural Britain: Using Q methodology to explore the views of first‐ and second‐generation women of Pakistaani origin. Midwifery 27: 458–468	15	In the 3–18 months ago	1st & 2nd generation Pakistani women	semi‐structured interview, urdu interpreter available	Q methodology ‐ mixed methods, thematic and factor analysis	Self‐identified in response to leaflet in child centre	None	10
8	Davies, M.M. Bath, P.A. (2001) The maternity information concerns of Somali women in the United Kingdom. Journal of Advanced Nursing; 36: 2: 237–245	13	Unknown	Somali women in the UK less than 10 years	Semi‐structured interview and focus group discussion with a translator	Grounded Theory	Invited by a community health worker and project interpreter	Yes	8
9	Finigan, V. Long, T. (2014) Skin‐to‐skin contact: multicultural perspectives on birth fluids and birth ‘dirt’. International Nursing Review 61: 270–277	20	1–2 days	English, Pakistani, Bangladeshi	Audiotaped diaries, semi‐structured interviews, photographs and video recordings with NHS translation	Phenomenology	Unclear	Yes	5
10	Goodwin, L. Hunter, B. Jones, A. (2017) The midwife‐woman relationship in a South Wales community: Experiences of midwives and migrant Pakistani women in early pregnancy. Health Expectations 21: 347–357	9	Still pregnant	Pakistani	Observation and interview, Interpreter offered	Ethnography	Recruited by MWs at Antenatal clinic	None	10
11	Harper Bulman, K. McCourt, C. (2002) Somali refugee women's experiences of maternity care in west London: A case study. Critical Public Health, 12:4, 365–380, DOI:10.1080/0958159021000029568	12	Still pregnant + previous children	Somali	Int and focus group in Somali in women's house with an interpreter, findings discussed with women	Phenomenology/action research/not clear	Snowball	None	8
12	Hassan, S.M. Leavey, C. Rooney, J.S. (2019) Exploring English speaking Muslim women's first‐time maternity experiences: a qualitative longitudinal interview study. BMC Pregnancy & Childbirth 19: 156 doi: 10.1186/s12884‐019‐2302‐y	7	Still pregnancy	Muslim women from Yemen, Somalia, India +2 white British women	3 in‐depth interviews with each woman, Antenataly, 2 and 4 months postnatally, in woman's home by the lead author in English	Qualitative longitudinal	Through women's groups	None	5
13	Jayaweera, H. D'Souza, L. Garcia, J. (2005) A local study of childbearing Bangladeshi women in the UK. Midwifery 21: 84–95	9	Pregnant or in 1 year of giving birth	Low‐income Bangladeshi women in Leeds	9 semi‐st interviews with women up to 1 year postpartum, 3 in woman's home by a researcher, 6 in Sylehti by org staff at the neighbourhood centre	Nested interview study principles of grounded theory	Approached by staff at family centres	None	7
14	Jomeen, J. Redshaw, M. (2013) Ethnic minority women's experience of maternity services in England. Ethnicity and Health, 18:3, 280–296	219	3 months ago	Women identified as BME on ONS record	Text responses to open questions in UK wide survey in English	Thematic analysis	ONS Survey text responses	Partly	9
15	Lam, E. Wittkowski, A. Fox, J.R.E. (2012) A qualitative study of the postpartum experience of Chinese women living in England. Journal of Reproductive and Infant Psychology 30:1: 105–119	8	In 1 year	Chinese	In‐depth interview in Chinese or English by researcher	Grounded theory	Poster at community centre	Yes	9
16	McAree, T. McCourt, C. Beake, S. (2010) Perceptions of group practice midwifery from women living in an ethnically diverse setting. Evidence based midwifery. 8:3: 91–97	12	In 2–3 years	S. Asia, Somalia, Balkans	S‐St Int in women homes by bi‐lingual researcher 6 in English, 3 in Gujarati	Grounded Theory	By letter and phone to women using the maternity service in last year	Partly	9
17	McCourt, C. Pearce, A. (2000) Does continuity of carer matter to women from minority ethnic groups? Midwifery 16:145–154	26	In 3–9 months	Caribbean, African, South and East Asian, Mediterranean, Middle Eastern women	Narrative Interview in womans home, 1 with somali translator	Principles of grounded theory	Follow‐up of survey non‐responders	None	10
18	McFadden, A. Siebelt, L. Jackson, C. Jones, H. Innes, N. MacGillivray, S. (2018) Enhancing Gypsy, Roma and Traveller peoples' trust. Report: University of Dundee	42	Unknown	Romany Gypsy, Irish Traveller and Eastern European Roma communities	4 case studies with an interpreter	Thematic analysis	Through charities	None	9
19	Moxey, J.M. Jones, L.L. (2016) A qualitative study exloring how Somali women exposed to female genital mutilation experience and perceive antenatal and intrapartum care in England. BMJ Open 6:e009846	10	In 5 years	Somali women, BMH	semi‐structured interview with a lay interpreter, known to the community, in an antenatal clinic	Descriptive qualitative but iterative rather than the framework	Snowball and convenience through community outreach workers	Yes	9
20	Ockleford EM; Berryman JC; Hsu R. (2004) Postnatal care: what new mothers say. British Journal of Midwifery. 12:3: 166–170	18	In 3 months	White and Indian women	Semi‐structured interview by Gujarati speaker in woman's home	Content analysis	Recruited at booking visit with the midwife	None	6
21	Phillimore, J. (2016) Migrant maternity in an era of superdiversity: New migrants' access to, and experience of, antenatal care in the West Midlands, UK. Social Science and Medicine 148: 152–159	82	In 5 years	28 countries, including China, Iran, Pakistan and Poland	Mixed methods questionnaire +13 in‐depth interviews by poly‐lingual interviewers	Interpretive thematic analysis	Purposive from DoH database	None	9
22	Puttusery, S. Twamley, K. Macfarlane, A. Harding, S. Baron, M. (2010) ‘You need that tender loving care’: maternity care experiences and expectations of ethnic minority women born in the United Kingdom. Journal of Health Services Research and Policy 15:3:156–162	34	In 1 year	UK‐born mothers of Black Caribbean, Black African, Indian, Pakistani, Bangladeshi and Irish descent	Qual in‐depth interview by 2 researchers in woman's home in English	Grounded Theory	Through Midwives at Antenatal clinic	Partly	9
23	Watson, H. Soltani, H. (2019) Perinatal mental ill health: the experiences of women from ethnic minority groups.British Journal of Midwifery. 27:10: 642–648	51	Unknown	20 South Asian women, 31 from 14 different ethnic backgrounds, table of characteristics missing	Survey online and face to face in English	Mixed methods	Facebook	None	8
24	Wittkowski, A. Zumla, A. Glendenning, S. Fox, J.R.E. (2011) The experienceof postnatal depression in South Asian mothers living in Great Britain: a qualitative study, Journal of Reproductive and Infant Psychology, 29:5, 480–492	10	In 3 months	South Asian Mothers	Interview in English	Grounded theory	Health visitor and midwife recruited	Yes	9

Abbreviation: CASP, Critical Appraisal Skills Programme.

**TABLE 3:** Thematic map and theme contribution of papers

Analytical theme	Descriptive theme	Codes	Contributing paper
Power of the technocratic birthing system	Accountability to the institution rather than to the woman	Functional versus woman‐centred care	1, 3, 5, 6, 7, 9, 14, 16, 17, 20, 21, 22
		Decontextualizing women, ritualized care	1, 2, 4, 5, 6, 9, 10, 11, 12, 14, 19, 21
		Resource barriers	5, 6, 13, 19, 21
Communication failures	Avoidance of complexity	Inadequate information or signposting	1, 2, 5, 6, 7, 10, 13, 15, 16, 17, 19, 21, 22, 23, 24
		Compromised consent	1, 4, 5, 6, 7, 8, 11, 12, 13, 14, 17
		Language	1, 2, 6, 8, 11, 13, 15, 19, 23
		Interpreters	1, 2, 4, 5, 6, 7, 8, 11, 14, 17, 21
Mistreatment of women	Differential treatment	Discrimination and prejudice	1, 5, 6, 7, 10, 11, 12, 14, 17, 24
		Isolating	1, 5, 6, 14, 16, 17, 18, 20, 23
		Unable to speak up/not listened to	1, 2, 5, 6, 7, 16, 17, 18, 22
		Lack of care	5,14,16,17,19,22
		Unkind	6,7,8,11,14,16,17,24
Woman‐centred care as exceptional, not routine	When resources allow engagement with the complexity of women's lives	Targeted service	1,5,6,7
		Cultural safety	1,2,4,6,10,12,14,16,24
		Continuity/trust	2,3,5,6,7,10,14,16,17,18
		Non judgemental	3,4,5,6,7,11,13,14,16,17,18, 22,23

## 5 BIRTHING IN A TECHNOCRATIC SYSTEM

Women of Black, Asian and minority ethnicities reported care to be functional rather than supportive that the maternity system was task focused, and failed to treat them as a person (Ali, 2004; Davies & Bath, 2001; Harper Bulman & McCourt, 2002; McAree et al., 2010; McCourt & Pearce, 2000) engendering feelings of being processed rather than being cared for (Beake et al., 2013; Birthrights, 2020; Cardwell & Wainwright, 2019; Jomeen & Redshaw, 2013; McAree et al., 2010; Ockleford et al., 2004; Puttusery et al., 2010). Among the women who reported their care as fragmented and task focused, they also described feeling the system was unable to engage with the complexity of their lives, with healthcare professionals assuming that they had access to childcare or transport, for example, which impacted women's ability to attend appointments (Birthrights, 2020; Cardwell & Wainwright, 2019; Jayaweera et al., 2005; Moxey & Jones, 2016; Phillimore, 2016). They described feeling judged by healthcare providers when they requested support related to personal safety or resources during their antenatal appointments (Cardwell & Wainwright, 2019; Goodwin et al., 2017; Jayaweera et al., 2005; Phillimore, 2016) and noted that some healthcare providers made assumptions that all women had safe and stable housing (Birthrights, 2020; Cardwell & Wainwright, 2019; Phillimore, 2016). Access to financial resources (Ali, 2004; Birthrights, 2020; Cardwell & Wainwright, 2019; Jayaweera et al., 2005; Phillimore, 2016), employer or childcare support for appointments or labour (Phillimore, 2016) were concerns which they felt unable to discuss with their midwife (Alshawish et al., 2013; Birthrights, 2020; Phillimore, 2016).

Women noticed the impact of short staffing and high workloads on the delivery of care, such that interactions with midwives in the antenatal and postnatal periods were described as ‘rushed’ (Jomeen & Redshaw, 2013; McAree et al., 2010; Phillimore, 2016; Puttusery et al., 2010) or overly focused on measurement activities (Birthrights, 2020; Cardwell & Wainwright, 2019; Davies & Bath, 2001; Harper Bulman & McCourt, 2002; Jomeen & Redshaw, 2013; McAree et al., 2010; Puttusery et al., 2010).

Staff were often too busy to come when called and when they did eventually come they were annoyed! (Indian Mother, UK born, Jomeen & Redshaw, 2013).

This context impacted negatively as ethnic minority women reported not feeling in control or participating in decision‐making. As a result, they experienced birthing as impersonal and dissatisfying:

I would have my appointments made for me and each time I went they would check or take what they wanted, and then I would leave without understanding what they had done… I saw no special kindness. They would just do the job and go (Somali mother, Harper Bulman & McCourt, 2002).

It was the midwife …. She did not want to know. She had a set of things she wanted me to do and she did not want me to ask any questions. It did not matter that I speak English (South Asian mother, Davies & Bath, 2001).

This experience was exacerbated by lack of continuity of care which made building a trusting relationship difficult (Beake et al., 2013; Goodwin et al., 2017; Jomeen & Redshaw, 2013; McAree et al., 2010; Phillimore, 2016; Puttusery et al., 2010) and impacted negatively on the quality of communication (Birthrights, 2020; Cardwell & Wainwright, 2019; McAree et al., 2010; Puttusery et al., 2010) and women's confidence to attend follow‐ups (Phillimore, 2016). In one study (McCourt & Pearce, 2000), women reported multiple shift changes of staff attending the birth and several non‐essential staff or students present without the woman understanding why they were there or giving her consent, which reinforced the perception of care as technocratic.

## 6 COMMUNICATION FAILURES

Several communication failures were described by women as a consequence of knowledge assumptions by midwives and their avoidance of time consuming or potentially complex engagements. Navigating the UK maternity system was described as especially hard if different specialists were involved in care (Ali, 2004; Birthrights, 2020; Cardwell & Wainwright, 2019; Davies & Bath, 2001; Goodwin et al., 2017; Moxey & Jones, 2016; Phillimore, 2016) or women were not confident in English (Ali, 2004; Alshawish et al., 2013; Birthrights, 2020; Lam et al., 2012). Women felt midwives assumed they knew how to find antenatal classes (Ali, 2004, Birthrights, 2020, Cardwell & Wainwright, 2019, Moxey & Jones, 2016), and reliable information to prepare for parenthood (Ali, 2004; Cardwell & Wainwright, 2019; Davies & Bath, 2001; Lam et al., 2012; McAree et al., 2010; McCourt & Pearce, 2000; Moxey & Jones, 2016; Phillimore, 2016; Watson & Soltani, 2019). They felt health professionals did not have time to signpost key information (Ali, 2004; Cardwell & Wainwright, 2019; Davies & Bath, 2001; McCourt & Pearce, 2000; Wittkowski et al., 2011) and that they were left to ‘fend for themselves’ (Ali, 2004; Cardwell & Wainwright, 2019).

…they gave lots of paperwork which, to be honest, I do not even think I've read of it to this day. (Ethnic minority Mother, ethnicity not specified, Cardwell & Wainwright, 2019).

Some had been unaware of the available birth choices (Ali, 2004; Birthrights, 2020; McAree et al., 2010) about pain relief (McAree et al., 2010) or where to go for help if they had concerns (Ali, 2004, Alshawish, 2013; Cardwell & Wainwright, 2019, Davies & Bath, 2001, Goodwin et al., 2017, Jomeen & Redshaw, 2013, Phillimore, 2016, Puttusery et al., 2010, Watson & Soltani, 2019, Wittkowski et al., 2011). Some women said that they did not know how to write a birth plan and felt their midwife had not checked if they needed support (Birthrights, 2020; Cardwell & Wainwright, 2019; Hassan et al., 2019; Puttusery et al., 2010). However, when midwives did share information, it was well received and gave women the chance to voice their concerns (Watson & Soltani, 2019).

Women who were not confident in English often reported language barriers and interpretation challenges when trying to communicate with maternity staff (Ali, 2004; Alshawish et al., 2013; Binder‐Finnema et al., 2012; Birthrights, 2020; Cardwell & Wainwright, 2019; Cross‐Sudworth et al., 2011; Davies & Bath, 2001; Harper Bulman & McCourt, 2002; Jayaweera et al., 2005; Jomeen & Redshaw, 2013; Lam et al., 2012; McCourt & Pearce, 2000; Moxey & Jones, 2016; Phillimore, 2016; Puttusery et al., 2010; Watson & Soltani, 2019). Concerns about consent were highlighted. In some cases, women had undergone procedures without fully understanding their purpose or risks (Ali, 2004; Alshawish et al., 2013; Beake et al., 2013; Birthrights, 2020; Cardwell & Wainwright, 2019; Davies & Bath, 2001; Harper Bulman & McCourt, 2002; McAree et al., 2010).

I do not know what's going to happen so and I did not know when they do a sweep I did not know what that was. (Ethnic minority Mother, ethnicity not specified, Beake et al., 2013).

Health‐trained interpreters were rarely used, resulting in reliance on friends and families (Ali, 2004; Alshawish et al., 2013; Binder‐Finnema et al., 2012; Cardwell & Wainwright, 2019; Cross‐Sudworth et al., 2011; Harper Bulman & McCourt, 2002; McCourt & Pearce, 2000; Phillimore, 2016), which caused discomfort and prevented full disclosure of symptoms (Ali, 2004; Alshawish et al., 2013; Binder‐Finnema et al., 2012; Cardwell & Wainwright, 2019; Davies & Bath, 2001; Harper Bulman & McCourt, 2002; Moxey & Jones, 2016), asking of questions (Birthrights, 2020; Cardwell & Wainwright, 2019; Davies & Bath, 2001; Harper Bulman & McCourt, 2002; McCourt & Pearce, 2000) or attendance at appointments (Birthrights, 2020; Harper Bulman & McCourt, 2002; Jayaweera et al., 2005; Phillimore, 2016). Women were not always aware of their right to a trained interpreter (Binder‐Finnema et al., 2012; Birthrights, 2020; Cross‐Sudworth et al., 2011). If an interpreter was present, women worried about the accuracy of translation and confidentiality (Ali, 2004; Davies & Bath, 2001; Harper Bulman & McCourt, 2002; Phillimore, 2016). Some women felt they were made responsible for finding or booking an interpreter (Ali, 2004, Alshawish, 2013, Birthrights, 2020, Davies & Bath, 2001, Harper Bulman & McCourt, 2002). In one study, women found the available translated information sheets unintelligible (Phillimore, 2016).

## 7 MISTREATMENT OF WOMEN

Differential treatment by staff impacted the quality of care women received (Ali, 2004; Birthrights, 2020; Cardwell & Wainwright, 2019; Goodwin et al., 2017; Harper Bulman & McCourt, 2002; Jomeen & Redshaw, 2013; McAree et al., 2010; McCourt & Pearce, 2000; McFadden et al., 2018; Ockleford et al., 2004; Wittkowski et al., 2011). Some women reported prejudice or discrimination based on their ethnicity, religion or culture (Ali, 2004; Birthrights, 2020; Cardwell & Wainwright, 2019; Davies & Bath, 2001; Goodwin et al., 2017) and being treated in an unsympathetic or unhelpful way, especially compared with the ways White mothers were seen to be treated (Ali, 2004, Beake et al., 2013, Birthrights, 2020, Cardwell & Wainwright, 2019, Cross‐Sudworth et al., 2011, Jomeen & Redshaw, 2013, McCourt & Pearce, 2000, McFadden et al., 2018,Wittkowski et al., 2011).

I was on a ward of four white women and I asked her, ‘You were really good with the person next door, could you help me. I asked two or three times I wanted to breastfeed and they did not come to me, yet they helped all the white women. (Muslim Mother, ethnicity not specified, Ali, 2004).

Direct discrimination, stereotyping or racist comments (Ali, 2004; Birthrights, 2020; Cardwell & Wainwright, 2019; Cross‐Sudworth et al., 2011; Goodwin et al., 2017; Harper Bulman & McCourt, 2002; Hassan et al., 2019; Jomeen & Redshaw, 2013; McCourt & Pearce, 2000) were noted, including the suggestion that Asian women made a fuss and were unable to tolerate pain (McCourt & Pearce, 2000), and that Bangladeshi women had children to get more state benefits (Binder‐Finnema et al., 2012; Wittkowski et al., 2011). Two studies reported that women felt coerced to have intra‐uterine devices fitted immediately after birth to control their fertility (Ali, 2004; McCourt & Pearce, 2000).

These experiences and a lack of support in hospital (Ali, 2004; Beake et al., 2013; Birthrights, 2020; Cardwell & Wainwright, 2019; Jomeen & Redshaw, 2013; McAree et al., 2010; Puttusery et al., 2010; Watson & Soltani, 2019) led women to feel isolated, abandoned (Ali, 2004; Birthrights, 2020; Cardwell & Wainwright, 2019; Jomeen & Redshaw, 2013; McAree et al., 2010; McFadden et al., 2018; Moxey & Jones, 2016; Ockleford et al., 2004; Puttusery et al., 2010; Wittkowski et al., 2011) and lonely (Birthrights, 2020; Davies & Bath, 2001). They felt afraid to ask questions or voice their concerns (Birthrights, 2020; Phillimore, 2016).

The fact you are asking for help, sometimes you are labelled, fear of, that you cannot cope…. You'll be judged. You're feeling like you cannot look after your baby. (Mother, ethnicity not specified, Cardwell & Wainright 2019).

Women whose children had been removed for safeguarding reasons said that they received no support from midwives and wished for more openness, honesty and bereavement support (Cardwell & Wainwright, 2019):

To me it was all just about taking the baby you know, really. They never really asked, really, you know, me being sad… (Mother, ethnicity not specified, Cardwell & Wainright 2019).

If women did ask for help or support, they felt ignored or that their request was an imposition (Ali, 2004; Birthrights, 2020; Cross‐Sudworth et al., 2011; Davies & Bath, 2001; Hassan et al., 2019; Jomeen & Redshaw, 2013; McAree et al., 2010; McCourt & Pearce, 2000; Puttusery et al., 2010; Watson & Soltani, 2019; Wittkowski et al., 2011). This was worse if there was a language barrier and made women feel frightened (Birthrights, 2020; Cardwell & Wainwright, 2019; Harper Bulman & McCourt, 2002; McAree et al., 2010; Phillimore, 2016). Even women who spoke English fluently recalled dismissive and disrespectful attitudes of maternity care staff, which discouraged them from speaking up (Ali, 2004; Binder‐Finnema et al., 2012; Birthrights, 2020; Cardwell & Wainwright, 2019; Cross‐Sudworth et al., 2011; Davies & Bath, 2001; Jomeen & Redshaw, 2013; McAree et al., 2010; McCourt & Pearce, 2000; Puttusery et al., 2010). Three papers reported women being denied adequate pain relief during labour despite asking for more (Birthrights, 2020; Jomeen & Redshaw, 2013; McCourt & Pearce, 2000). In addition to making women feel low or scared (Jomeen & Redshaw, 2013; McAree et al., 2010; McCourt & Pearce, 2000; Puttusery et al., 2010; Wittkowski et al., 2011), the uncaring behaviour by the staff made them cautious about engaging with the hospital maternity service in future pregnancies Cardwell & Wainwright, 2019, McCourt & Pearce, 2000, Puttusery et al., 2010).

I will not have a baby in a hospital again… It's not worth going to hospital because the experience I had was just terrible (African first‐time mother, Puttusery et al., 2010).

One group of women who had experienced genital cutting (Gillespie, 2012) described how a lack of understanding of the practice among attending staff had negatively impacted their birth experience (Harper Bulman & McCourt, 2002). Another study described Muslim women being ‘told off’ for fasting during Ramadan, being interrogated for refusing fetal screening, and not being informed that the vitamin K injection given to newborns contains porcine ingredients (Hassan et al., 2019). These findings are included for completeness but are reported with low confidence due to the single paper containing this evidence, which reports from a sample of seven women with no reflexivity and poor reporting of the method. Requests for female birth attendants by Muslim women and their partners were not always accommodated, causing them to feel guilty, and prompting decisions to birth at home or change healthcare providers (Ali, 2004; Alshawish et al., 2013; Birthrights, 2020; Hassan et al., 2019; Jomeen & Redshaw, 2013).

In two papers, women reported that staff audibly discussed sensitive personal information about them standing just behind a curtain in an open ward (Birthrights, 2020; Cardwell & Wainwright, 2019). Privacy was also an issue: women felt disrespected when staff would keep opening their bed curtains which they had closed to breastfeed or pray (Ali, 2004; Birthrights, 2020; Hassan et al., 2019).

## 8 WOMAN‐CENTRED CARE AS EXCEPTIONAL, NOT ROUTINE

Despite the emphasis on task‐focused care in a technocratic system and evidence of mistreatment of women, the synthesis also provided some examples of positive experiences of care. Sadly, these were often the exception and were confined to specific continuity of care services. Woman‐centred care was not the norm, nor was it reflected in the experiences of the majority of women in this synthesis. Where such care was delivered, it resulted in positive experiences (Beake et al., 2013, Birthrights, 2020, Cardwell & Wainwright 2019, Goodwin et al., 2017, McAree et al., 2010, McCourt & Pearce, 2000, McFadden et al., 2018, Moxey & Jones, 2016, Watson & Soltani, 2019) and fostered a sense of control (McCourt & Pearce, 2000), irrespective of whether the midwife and the woman shared ethnic or racial identity (Binder‐Finnema et al., 2012; Cardwell & Wainwright, 2019; Puttusery et al., 2010). Trusting relationships with maternity staff made women feel safe, and reassured (McAree et al., 2010; McCourt & Pearce, 2000; McFadden et al., 2018) and improved access to information for the woman and her family (Birthrights, 2020; Goodwin et al., 2017; McAree et al., 2010; Watson & Soltani, 2019).

So I used to get along with her so good I used to talk to her about everything that I did not even speak to my husband or mum about …. You know when you get to know someone, it's easier to talk and stuff (Ethnic minority mother, ethnicity not specified, Beake et al., 2013).

Such relationships gave women confidence to ask questions and share decision‐making (Binder‐Finnema et al., 2012; McCourt & Pearce, 2000; Moxey & Jones, 2016).

My voice was heard, you know, they took my issues to heart. (Mother involved with social services, ethnicity not specified, Cardwell & Wainwright, 2019).

These relationships also facilitated self‐efficacy (Birthrights, 2020; Cross‐Sudworth et al., 2011; Jomeen & Redshaw, 2013; McCourt & Pearce, 2000). Trust was built more easily when the woman had the same midwife looking after her throughout pregnancy and labour (Beake et al., 2013; Birthrights, 2020; Cardwell & Wainwright, 2019; Cross‐Sudworth et al., 2011; McAree et al., 2010; McCourt & Pearce, 2000; McFadden et al., 2018; Moxey & Jones, 2016) who knew her and her family and did not require a fresh set of explanations on every visit (Beake et al., 2013; Cardwell & Wainwright, 2019; McAree et al., 2010; McCourt & Pearce, 2000; McFadden et al., 2018). Women felt that continuity made communication easier (Beake et al., 2013; Birthrights, 2020; Cardwell & Wainwright, 2019; McAree et al., 2010; McCourt & Pearce, 2000; Watson & Soltani, 2019), made them feel cared for (Beake et al., 2013; Birthrights, 2020; Cardwell & Wainwright, 2019; Cross‐Sudworth et al., 2011; Harper Bulman & McCourt, 2002; Jomeen & Redshaw, 2013; McFadden et al., 2018), not judged (Cardwell & Wainwright, 2019; Cross‐Sudworth et al., 2011; Harper Bulman & McCourt, 2002; McCourt & Pearce, 2000; Puttusery et al., 2010), well supported (Beake et al., 2013; Binder‐Finnema et al., 2012; Birthrights, 2020; Cardwell & Wainwright, 2019; Harper Bulman & McCourt, 2002; Jomeen & Redshaw, 2013; McFadden et al., 2018), and listened to (Birthrights, 2020; Cardwell & Wainwright, 2019; Cross‐Sudworth et al., 2011; Harper Bulman & McCourt, 2002; McCourt & Pearce, 2000; Watson & Soltani, 2019). Women appreciated when midwives were sensitive to their cultural and religious beliefs (Ali, 2004; Moxey & Jones, 2016) and Muslim parents valued the information given in classes on how to fulfil religious observance during pregnancy and birth (Ali, 2004).

Some papers described targeted services that offered additional support to ethnic minority women (Ali, 2004; Birthrights, 2020; Cardwell & Wainwright, 2019; Jayaweera et al., 2005; McAree et al., 2010), but referral to these charities and mental health services appeared highly dependent on local knowledge of the midwife (Birthrights, 2020; Cardwell & Wainwright, 2019; Cross‐Sudworth et al., 2011).

## 9 DISCUSSION

### 9.1 Main findings

Our synthesis of 24 qualitative studies highlights how the technocratic birthing system and discriminatory practices in the UK NHS maternity services fail ethnic minority women. In the context of chronic understaffing and heavy workloads, there is a focus on measurements and procedures rather than the provision of a kind, holistic women‐centred care. Ethnic minority women are being left in the dark about what to expect, their rights and their choices, during pregnancy, birth and postnatally. Particular communication failures, due to a woman's limited English or cultural customs unfamiliar to maternity staff, may be symptoms of an overstretched workforce or manifestations of a deeper and generalized tendency to undermine and silence ethnic minority women in maternity care. Evidence of more direct forms of discrimination based on race and religion (and their intersections with economic and social disadvantages) suggest a perverse inversion, whereby women in need of the greatest support are likely to receive the least. Woman‐centred continuity of care models resulted in positive experiences, but this was often only found in pockets with the personnel and mandate rather than being a norm across service provision.

### 9.2 Strengths and limitations

To our knowledge, this is the first attempt to synthesize the qualitative literature from the UK exploring ethnic minority women's experiences of maternity services and we have used rigorous methods (Atkins et al., 2008; CASP, 2019; Karnieli‐Miller et al., 2009) for synthesizing and assessing the quality of the studies included.

Evidence synthesis works with secondary data and is limited by the original research questions of the papers, the quality of their methods and the presentation of their findings. Our search methods returned charity research reports and policy‐oriented papers, publication of doctoral research with local communities, alongside academic papers from research institutions. This variety meant that there was variation in rigour and reporting of methods, and sometimes a failure to consider power in the researcher–participant relationship. Notwithstanding these differences, our involved inductive approach has an added value by integrating these different sources to reveal new themes of interest.

### 9.3 Interpretation (findings in light of other evidence)

This synthesis augments the MBRRACE (Knight et al., 2020) analysis to illustrate how systemic biases, perpetuated by staff without skills and knowledge to understand the needs or listen to the concerns of ethnic minority women can prevent those women most at risk from receiving the care they need (Esegbona‐Adeigbe, 2021). Over 90% of the 566 deaths in the MBRRACE analysis were of women with a combination of risk factors that were also mentioned in this synthesis, reinforcing that ethnic minority women are at risk of poorer care and mistreatment. Core concepts identified as necessary for a positive birth experience among all birthing women include respectful care, trusting relationships, control and participation in decision‐making (Downe et al., 2018; Karlsdottir et al., 2018; Renfrew et al., 2014). These concepts were also highlighted as necessary for the ethnic minority women who participated in the studies included in our synthesis. However, they are entering the system already facing enormous disadvantages due to structural racism in society, and then there is further damage by more direct forms of racism in maternity care, both of which are not faced by white majority women.

Complaints of a dissatisfying birth experience in this underfunded, overstretched technocratic birthing system are shared by the white majority (Davis‐Floyd, 2003; Reed et al., 2017; Scamell & Aleszewski, 2012; Walsh, 2010). The technocratic approach, where clinical tasks and the safety agenda are privileged over person‐centred care, has been linked with negative psychological and social consequences (Beck, 2011; Benoit et al., 2010; Forssen, 2012; Reed et al., 2017; Soet et al., 2003). This has stimulated the Continuity of Midwifery Carer (CMC) policy linked to the Better Births maternity review (Department of Health, 2017) and aims to have at least 35% of birthing women receiving CMC by 2025 (NHSE, 2019). In light of the growing awareness of the greater impact of this system on ethnic minority women in the mortality and morbidity statistics reported by MBRRACE, CMC models have since been targeted to women of Black, Asian and minority ethnic backgrounds with the realization that they could deliver significant improvements in care experiences (NHSE, 2019). The papers reviewed for this synthesis were before this service change.

However, this is predicated on work to recruit and retain midwives, which is an ongoing concern (Hall, 2021), to reduce the staffing pressures known to engender a routinized, technocratic model of practice (Kirkup, 2021). Writing about UK mental health services, Nazroo et al. (2020) consider how, to tolerate the circumstances created by resource constraints, commissioners and care providers may feel the need to distance themselves from those receiving care. This is much easier when providing care to particular groups that can be treated as ‘other’ (such as racialized groups). This ‘othering’ creates and sustains inequalities, such that unequal health outcomes are understood simply as a reflection of wider structural conditions, in the context of resource constraints, and easily accepted as the norm.

Many women represented in the papers included in this synthesis reported instances of bullying, discrimination, unconsented procedures, coercive or insensitive care. Sometimes this was through omission or lack of consideration, but at others was direct. The recently proposed National Institute of Clinical Excellence (NICE) guideline recommending that Black, Asian and ethnic minority women be offered clinical induction at 39 weeks, acknowledges the excess risks for ethnic minority women but fails to articulate where the risk lies. Given mounting evidence from the UK of racism in healthcare, and in maternity care, in particular, this silence is significant and reinforces the false medical narrative that pathologizes ethnic minority bodies as deficient or inferior. In 2020, The American Medical Association explicitly recognized racism as a threat to public health and, in 2021, adopted new guidelines to confront systemic racism in medicine (American Medical Association [AMA], 2021). A similar move is urgently needed in the UK.

The experiences reported in this synthesis, alongside the disparities highlighted in the MBRRACE report (Knight et al., 2020), add context to the legal charity ‘Birthrights’ national inquiry into disparities in maternity care experiences and the impact of systemic racism (Birthrights, 2021). While acknowledging, this may not be the experience of all ethnic minority women, this synthesis uncovers the presence of racism in UK maternity services. It documents the impact that this has on the experience and well‐being of birthing women from ethnic minorities and is thus a necessary core consideration in a woman‐centred care agenda (Trepagnier, 2017).

## 10 CONCLUSION

This synthesis shows that ethnic minority women report positive pregnancy and birth experiences when they are in receipt of kind, respectful and woman‐centred midwifery care. However, these experiences are often the exception in the overstretched technocratic birthing system of the UK. The integration of these 24 studies reveals varied and disturbing forms of mistreatment and poor care for ethnic minority women in the UK and it seems probably that these differences in experience are linked to the inequalities in outcomes identified in the MBRRACE report. There is clearly much to be done in education and practice to address these concerns and improve these women's experience of the birthing journey.

## CONFLICT OF INTEREST

The authors report no financial or personal interests.

## AUTHOR CONTRIBUTIONS

Jennifer MacLellan conceived the study. Jennifer MacLellan and Sarah Collins designed and performed the search, study screening, data extraction and preliminary data analysis in duplicate. Tanvi Rai was the arbiter for unresolved conflicts. Jennifer MacLellan, Sarah Collins, Margaret Myatt, Wanja Knighton and Tanvi Rai refined the analysis. Jennifer MacLellan, Sarah Collins and Tanvi Rai wrote the manuscript with revisions based on Catherine Pope Wanja Knighton and Margaret Myatt comments. All authors accept responsibility for the manuscript.

## DETAILS OF ETHICS APPROVAL

Ethics approval was not required for secondary use of data in this systematic evidence synthesis. This manuscript is an honest, accurate and transparent account of the study being reported that no important aspects of the study have been omitted and that any discrepancies from the study as planned have been explained. Data extracted from included studies and data used for all analyses are available from the corresponding author on request.

### OPEN RESEARCH BADGES

This article has a preregistered research design available at https://www.crd.york.ac.uk/prospero/display_record.php?ID=CRD42020225758

### PEER REVIEW

The peer review history for this article is available at https://publons.com/publon/10.1111/jan.15233.

## Supporting information


DataS 1

FigureS 1

## Data Availability

Data sharing not applicable to this article as no datasets were generated or analysed during the current study.

## References

[jan15233-bib-0001] Ali, N. (2004). Experiences of maternity services: Muslim Women's perspectives. The Maternity Alliance. https://www.maternityaction.org.uk/wp

[jan15233-bib-0002] Alshawish, E. , Marsden, J. , Yeowell, G. , & Wibberley, C. (2013). Investigating access to and use of maternity health‐care services in the UK by Palestinian women. British Journal of Midwifery, 21, 571–577.

[jan15233-bib-0003] American Medical Association (AMA) AMA adopts guidelines that confront systemic racism in medicine. Press release. 2021. https://www.ama‐assn.org/press‐center/press‐releases/ama‐adopts‐guidelines‐confront‐systemic‐racism‐medicine

[jan15233-bib-0004] Aquino, M. R. , Edge, D. , & Smith, D. M. (2015). Pregnancy as an ideal time for intervention to address the complex needs of black and minority ethnic women: Views of British midwives. Midwifery, 31, 373–379. 10.1016/j.midw.2014.11.006 25483209

[jan15233-bib-0005] Atkins, S. , Lewin, S. , Smith, H. , Engel, M. , Fretheim, A. , & Volmink, J. (2008). Conducting a meta‐ethnography of qualitative literature: Lessons learnt. BMC Medical Research Methodology, 8, 21. 10.1186/1471-2288-8-21 18416812 PMC2374791

[jan15233-bib-0006] Beake, S. , Acosta, L. , Cooke, P. , & McCourt, C. (2013). Caseload midwifery in a multi‐ethnic community: The woman's experiences. Midwifery, 29, 996–1002. 23415359 10.1016/j.midw.2013.01.003

[jan15233-bib-0007] Beck, C. T. (2011). A metaethnography of traumatic childbirth and its aftermath: Amplified causal looping. Qualitative Health Research, 21, 301–311. 21131566 10.1177/1049732310390698

[jan15233-bib-0008] Benoit, C. , Zadoroznyj, M. , Hallgrimsdottir, H. , Treloar, A. , & Taylor, K. (2010). Medical dominance and neoliberalisation in maternal care provision: The evidence from Canada and Australia (pp. 475–481). Social Science and Medicine. 10.1016/j.socscimed.2010.04.005 PMC4445451 20570030

[jan15233-bib-0009] Binder‐Finnema, P. , Borne, Y. , Johnsdotter, S. , & Essen, B. (2012). Shared language is essential: Communication in a multiethnicity obstetric care setting. Journal of Health Communication, 2012, 1171–1186. 10.1080/10810730.2012.665421 22703624

[jan15233-bib-0010] Birthrights . (2020). Holding it all together: Understanding how far the human rights of women facing disadvantage are respected during pregnancy, birth, postnatal care. Birthrights. https://www.birthrights.org.uk/wp‐content/uploads/2019/09/Holding‐it‐all‐together‐Full‐report‐FINAL‐Action‐Plan.pdf

[jan15233-bib-0011] Birthrights (2021) Racial injustice in maternity care. A human rights inquiry: Call for evidence Birthrights. https://www.birthrights.org.uk/campaigns‐research/racial‐injustice/

[jan15233-bib-0012] Cardwell, V. , & Wainwright, L. (2019). Making better births a reality for women with multiple disadvantages. A qualitative peer research study exploring perinatal women's experiences of care and services in north—East London. Birth Companions and Revolving Doors Agency.

[jan15233-bib-0013] Critical Appraisal Skills Programme CASP CHECKLISTS ‐ CASP ‐ Critical appraisal skills programme. 2019. casp‐uk.net

[jan15233-bib-0014] Cross‐Sudworth, F. , Williams, A. , & Herron‐Marx, S. (2011). Maternity services in multi‐cultural Britain: Using Q methodology to explore the views of first‐ and second ‐ generation women of Pakistani origin. Midwifery, 27, 458–468. 21036439 10.1016/j.midw.2010.03.001

[jan15233-bib-0015] Davies, M. M. , & Bath, P. A. (2001). The maternity information concerns of Somali women in the United Kingdom. Journal of Advanced Nursing, 36(2), 237–245. 11580798 10.1046/j.1365-2648.2001.01964.x

[jan15233-bib-0016] Davis, D. (2019). Obstetric racism: The racial politics of pregnancy, labor, and birthing. Medical Anthropology, 38, 560–573. 10.1080/01459740.2018.1549389 30521376

[jan15233-bib-0017] Davis‐Floyd, R. E. (2003). Birth as an American rite of passage. University of California Press.

[jan15233-bib-0018] Department of Health . (1993). Changing childbirth: Reports of the expert maternity group parts 1 & 2. Stationary Office.

[jan15233-bib-0019] Department of Health . (1997). The new NHS: Modern and dependable. HMSO; Stationary Office.

[jan15233-bib-0020] Department of Health . (2017). Better births: Improving outcomes of maternity services in England – A five year forward view for maternity care. Stationary Office.

[jan15233-bib-0021] Downe, S. , Lawrie, T. A. , Finlayson, K. , & Oladapo, O. (2018). Effectiveness of respectful care policies for women using routine intrapartum services: A systematic review. Reproductive Health, 15, 1. 10.1186/s12978-018-0466-y 29409519 PMC5801845

[jan15233-bib-0022] Esegbona‐Adeigbe, S. (2021). The impact of a Eurocentric curriculum on racial disparities in maternal health. European Journal of Midwifery, 5, 36. 10.18332/ejm/140086 34568777 PMC8419870

[jan15233-bib-0023] Finigan, V. , & Long, T. (2014). Skin‐to‐skin contact: Multicultural perspectives on birth fluids and birth ‘dirt’. International Nursing Review, 61, 270–277. 24712443 10.1111/inr.12100 PMC4265244

[jan15233-bib-0024] Fisher, R. , & Fraser, C. (2020). Who gets in? What does the 2020 GP patient survey tell us about access to general practice? The Health Foundation. https://www.health.org.uk/news‐and‐comment/charts‐and‐infographics/who‐gets‐in

[jan15233-bib-0025] Forssen, A. S. K. (2012). Lifelong significance of disempowering experiences in prenatal and maternity care: Interviews with elderly Swedish women. Qualitative Health Research, 22, 1535–1546. 22745366 10.1177/1049732312449212

[jan15233-bib-0026] Gillespie G . Why do we use the term female genital cutting and not female genital mutilation. The Orchid Project. 2012. https://www.orchidproject.org/why‐do‐we‐use‐the‐term‐female‐genital‐cutting‐and‐not‐female‐genital‐mutilation/

[jan15233-bib-0027] Goodwin, L. , Hunter, B. , & Jones, A. (2017). The midwife‐woman relationship in a South Wales community: Experiences of midwives and migrant Pakistani women in early pregnancy. Health Expectations, 21, 347–357. 28960699 10.1111/hex.12629 PMC5750740

[jan15233-bib-0028] Hall, J. (2021). Midwifery staff shortages – Have we reached tipping point?. Maternity and Midwifery Forum Blog. https://www.maternityandmidwifery.co.uk/midwifery‐staff‐shortages‐have‐we‐reached‐tipping‐point/

[jan15233-bib-0029] Harper Bulman, K. , & McCourt, C. (2002). Somali refugee women's experiences of maternity care in West London: A case study. Critical Public Health, 12, 365–380. 10.1080/0958159021000029568

[jan15233-bib-0030] Hassan, S. M. , Leavey, C. , & Rooney, J. S. (2019). Exploring English speaking Muslim women's first‐time maternity experiences: A qualitative longitudinal interview study. BMC Pregnancy & Childbirth, 19, 156. 10.1186/s12884-019-2302-y 31060520 PMC6501380

[jan15233-bib-0031] Henderson, J. , Gao, H. , & Redshaw, M. (2018). Experiencing maternity care: The care received and perceptions of women from different ethnic groups. BMC Pregnancy and Childbirth, 2013, 196. http://www.biomedcentral.com/1471‐2393/13/196 10.1186/1471-2393-13-196 PMC3854085 24148317

[jan15233-bib-0032] Hoffman, K. M. , Trawalter, S. , Axt, J. R. , & Oliver, M. N. (2016). Racial bias in pain assessment and treatment recommendations, and false beliefs about biological differences between blacks and whites. Proceedings of the National Academy of Sciences of the United States of America, 113, 4296–4301. 27044069 10.1073/pnas.1516047113 PMC4843483

[jan15233-bib-0033] Jayaweera, H. , D'Souza, L. , & Garcia, J. (2005). A local study of childbearing Bangladeshi women in the UK. Midwifery, 21, 84–95. 15740820 10.1016/j.midw.2004.09.003

[jan15233-bib-0034] Jomeen, J. , & Redshaw, M. (2013). Ethnic minority women's experience of maternity services in England. Ethnicity and Health, 18, 280–296. 10.1080/13557858.2012.730608 23039872

[jan15233-bib-0035] Karlsdottir, S. L. , Sveinsdottir, H. , Kristjansdottir, H. , Aspelund, T. , & Olafsdottir, O. A. (2018). Predictors of women's positive childbirth pain experience: Findings from an Icelandic national study. Women and Birth, 31, e178–e184. 10.1016/j.wombi.2017.09.007 28943317

[jan15233-bib-0036] Karnieli‐Miller O , Strier R , Pessach L , (2009) Power relations in qualitative research Qualitative Health Research 2009;19:279‐89 10.1177/1049732308329306 19150890

[jan15233-bib-0037] Kirkup, B. (2021). EPE0005 – Expert panel: Evaluation of the Government's commitments in the area of maternity services in England. Written Evidence Health and Social Care Committee. https://committees.parliament.uk/committee/81/health‐and‐social‐care‐committee/publications/written‐evidence/?SearchTerm=bill+kirkup

[jan15233-bib-0038] Knight, M. , Bunch, K. , Tuffnell, D. , Shakespeare, J. , Kotnis, R. , Kenyon, S. , et al. (Eds.). (2020). MBRRACE‐UK: Saving lives, improving Mothers' Care 2020: Lessons to inform maternity care from the UKand Ireland confidential enquiries in maternal death and morbidity 2016–18. National Perinatal Epidemiology Unit, University of Oxford.

[jan15233-bib-0039] Lam, E. , Wittkowski, A. , & Fox, J. R. E. (2012). A qualitative study of the postpartum experience of Chinese women living in England. Journal of Reproductive and Infant Psychology, 30, 105–119. 10.1080/02646838.2011.649472

[jan15233-bib-0040] Lyons, S. M. , O'Keeffe, F. , Clarke, A. T. , & Staines, A. (2008). Cultural diversity in the Dublin maternity services: The experience of maternity service providers when caring for ethnic minority women. Ethnicity and Health, 13, 261–276. 18568976 10.1080/13557850801903020

[jan15233-bib-0041] MacLellan, J. , Collins, S. , Myatt, M. , Rai, T. , et al. (2020). A Metasynthesis of Black, Asian and minority ethnic women's experiences of maternity services in the UK (CRD42020225758 Available from:). PROSPERO. https://www.crd.york.ac.uk/prospero/display_record.php?ID=CRD42020225758 10.1111/jan.15233 PMC9314829 35332568

[jan15233-bib-0042] McAree, T. , McCourt, C. , & Beake, S. (2010). Perceptions of group practice midwifery from women living in an ethnically diverse setting. Evidence Based Midwifery, 8, 91–97.

[jan15233-bib-0043] McCourt, C. , & Pearce, A. (2000). Does continuity of carer matter to women from minority ethnic groups? Midwifery, 16, 145–154. 10.1054/midw.2000.0204 11151550

[jan15233-bib-0044] McFadden, A. , Siebelt, L. , Jackson, C. , Jones, H. , Innes, N. , MacGillivray, S. , & Gypsy, E. (2018). Roma and Traveller peoples' trust: Report. University of Dundee. 10.20933/100001117

[jan15233-bib-0045] Metzl, J. M. , & Hansen, H. (2014). Structural competency: Theorizing a new medical engagement with stigma and inequality. Social Science and Medicine, 103, 126–133. 10.1016/j.socscimed.2013.06.032 24507917 PMC4269606

[jan15233-bib-0046] Miller, S. A. (2001). PICO worksheet and search strategy. US National Center for Dental Hygiene Research.

[jan15233-bib-0047] Moxey, J. M. , & Jones, L. L. (2016). A qualitative study exploring how Somali women exposed to female genital mutilation experience and perceive antenatal and intrapartum care in England. BMJ Open, 6, e009846. 10.1136/bmjopen-2015-009846 PMC4716221 26743705

[jan15233-bib-0048] Murray, L. , Windsor, C. , Parker, E. , & Tewfik, O. (2010). The experiences of African women giving birth in Brisbane, Australia. Health Care for Women International, 31, 458–472. 10.1080/07399330903548928 20390666

[jan15233-bib-0049] Nazroo, J. Y. , Bhui, K. S. , & Rhodes, T. (2020). Where next for understanding race/ethnic inequalities in severe mental illness? Structural, interpersonal and institutional racism. Sociology of Health and Illness, 42(2), 262–276. 31562655 10.1111/1467-9566.13001 PMC7028120

[jan15233-bib-0050] NHSE . (2019). Targeted and enhanced midwifery‐led continuity of carer. NHS England. https://www.england.nhs.uk/ltphimenu/maternity/targeted‐and‐enhanced‐midwifery‐led‐continuity‐of‐carer/

[jan15233-bib-0051] Ockleford, E. M. , Berryman, J. C. , & Hsu, R. (2004). Postnatal care: What new mothers say. British Journal of Midwifery, 12(3), 166–170.

[jan15233-bib-0052] O'Mahony, J. , & Donnelly, T. (2010). Immigrant and refugee women's post‐partum depression help‐seeking experiences and access to care: A review and analysis of the literature. Journal of Psychiatry and Mental Health Nursing, 17, 917–928. 10.1111/j.1365-2850.2010.01625.x 21078007

[jan15233-bib-0053] Page, M. J. , McKenzie, J. E. , Bossuyt, P. M. , Boutron, I. , Hoffmann, T. C. , Mulrow, C. D. , et al. (2021). The PRISMA 2020 statement: An updated guideline for reporting systematic reviews. British Medical Journal, 372, n71. 10.1136/bmj.n71 33782057 PMC8005924

[jan15233-bib-0054] Phillimore, J. (2016). Migrant maternity in an era of superdiversity: New migrants' access to, and experience of, antenatal care in the west midlands, UK. Social Science and Medicine, 148, 152–159. 26705910 10.1016/j.socscimed.2015.11.030

[jan15233-bib-0055] Puttusery, S. , Twamley, K. , Harding, S. , Mirsky, J. , Baron, M. , & Macfarlane, A. (2008). ‘They're more like ordinary stroppy British women’: Attitudes and expectations of maternity care professionals to UK‐born ethnic minority women. Journal of Health Services Research & Policy, 13(4), 195–201. 18806176 10.1258/jhsrp.2008.007153

[jan15233-bib-0056] Puttusery, S. , Twamley, K. , Macfarlane, A. , Harding, S. , & Baron, M. (2010). ‘You need that tender loving care’: Maternity care experiences and expectations of ethnic minority women born in the United Kingdom. Journal of Health Services Research and Policy, 15(3), 156–162. 20466754 10.1258/jhsrp.2009.009067

[jan15233-bib-0057] Race Disparity Unit . Writing about ethnicity. 2021. https://www.ethnicity‐facts‐figures.service.gov.uk/style‐guide/writing‐about‐ethnicity

[jan15233-bib-0058] Rayment‐Jones, H. , Harris, J. , Harden, A. , Khan, Z. , & Sandall, J. (2019). How do women with social risk factors experience United Kingdom maternity care? A realist synthesis. Birth: Issues in Perinatal Care, 46, 461–474. 10.1111/birt.12446 PMC6771833 31385354

[jan15233-bib-0059] Redshaw, M. , & Heikkila, K. (2011). Ethnic differences in women's worries about labour and birth. Ethnicity and Health, 16(3), 213–223. 21500115 10.1080/13557858.2011.561302

[jan15233-bib-0060] Reed, R. , Sharman, R. , & Inglis, C. (2017). Women's descriptions of childbirth trauma relating to the care provider actions and interactions. BMC Pregnancy and Childbirth, 17, 21. 10.1186/s12884-016-1197-0 28068932 PMC5223347

[jan15233-bib-0061] Renfrew, M. J. , McFadden, A. , Bastos, M. H. , Campbell, J. , Channon, A. A. , Cheung, N. F. , Silva, D. R. A. D. , Downe, S. , Kennedy, H. P. , Malata, A. , McCormick, F. , Wick, L. , & Declercq, E. (2014). Midwifery and quality care: Findings from a new evidence‐informed framework for maternal and newborn care. The Lancet, 384(9948), 1129–1145. 10.1016/s0140-67369(14)607893 24965816

[jan15233-bib-0062] Rethlefsen, M. L. , Kirtley, S. , Waffenschmidt, S. , Ayala, A. P. , Moher, D. , et al. (2021). PRISMA‐S group. PRISMA‐S: An extension to the PRISMA statement for reporting literature searches in systematic reviews. Systematic Reviews, 10(1), 39. 34285662 10.5195/jmla.2021.962 PMC8270366

[jan15233-bib-0063] Scamell, M. , & Aleszewski, A. (2012). Fateful moments and the categorisation of risk: Midwifery practice and the ever narrowing window of normality during childbirth. Health, Risk and Society, 14(2), 207–221. 10.1080/13698575.2012.661041

[jan15233-bib-0064] Soet, J. E. , Brack, G. A. , & Dilorio, C. (2003). Prevalence and predictors of women's experiences of psychological trauma during childbirth. Birth, 30(1), 36–46. 12581038 10.1046/j.1523-536x.2003.00215.x

[jan15233-bib-0065] Thomas, J. , & Harden, A. (2008). Methods for the thematic synthesis of qualitative research in systematic reviews. BMC Medical Research Methodology, 8, 45. 10.1186/1471-2288-8-45 18616818 PMC2478656

[jan15233-bib-0066] Trepagnier, B. (2017). Silent racism: How well‐meaning white people perpetuate the racial divide. Routledge.

[jan15233-bib-0067] Walsh, D. (2010). Childbirth embodiment: Problematic aspects of current understandings. Sociology of Health and Illness, 32(3), 486–501. 20003040 10.1111/j.1467-9566.2009.01207.x

[jan15233-bib-0068] Watson, H. , & Soltani, H. (2019). Perinatal mental ill health: The experiences of women from ethnic minority groups. British Journal of Midwifery., 27(10), 642–648.

[jan15233-bib-0069] Wikberg, A. , Eriksson, K. , & Bondas, T. (2012). Intercultural caring from the perspectives of immigrant new mothers. Journal of Obstetric Gynecologic & Neonatal Nursing, 41, 5–649. 10.1111/j.1552-6909.2012.01395.x 22823102

[jan15233-bib-0070] Wittkowski, A. , Zumla, A. , Glendenning, S. , & Fox, J. R. E. (2011). The experience of postnatal depression in south Asian mothers living in Great Britain: A qualitative study. Journal of Reproductive and Infant Psychology, 29(5), 480–492. 10.1080/02646838.2011.639014

