# Supplementary figures and images for: Black, Asian and minority ethnic women's experiences of maternity services in the UK: A qualitative evidence synthesis
Source: J Adv Nurs. 2022 Mar 24;78(7):2175–90. doi: 10.1111/jan.15233 (PMC9314829; doi:10.1111/jan.15233)

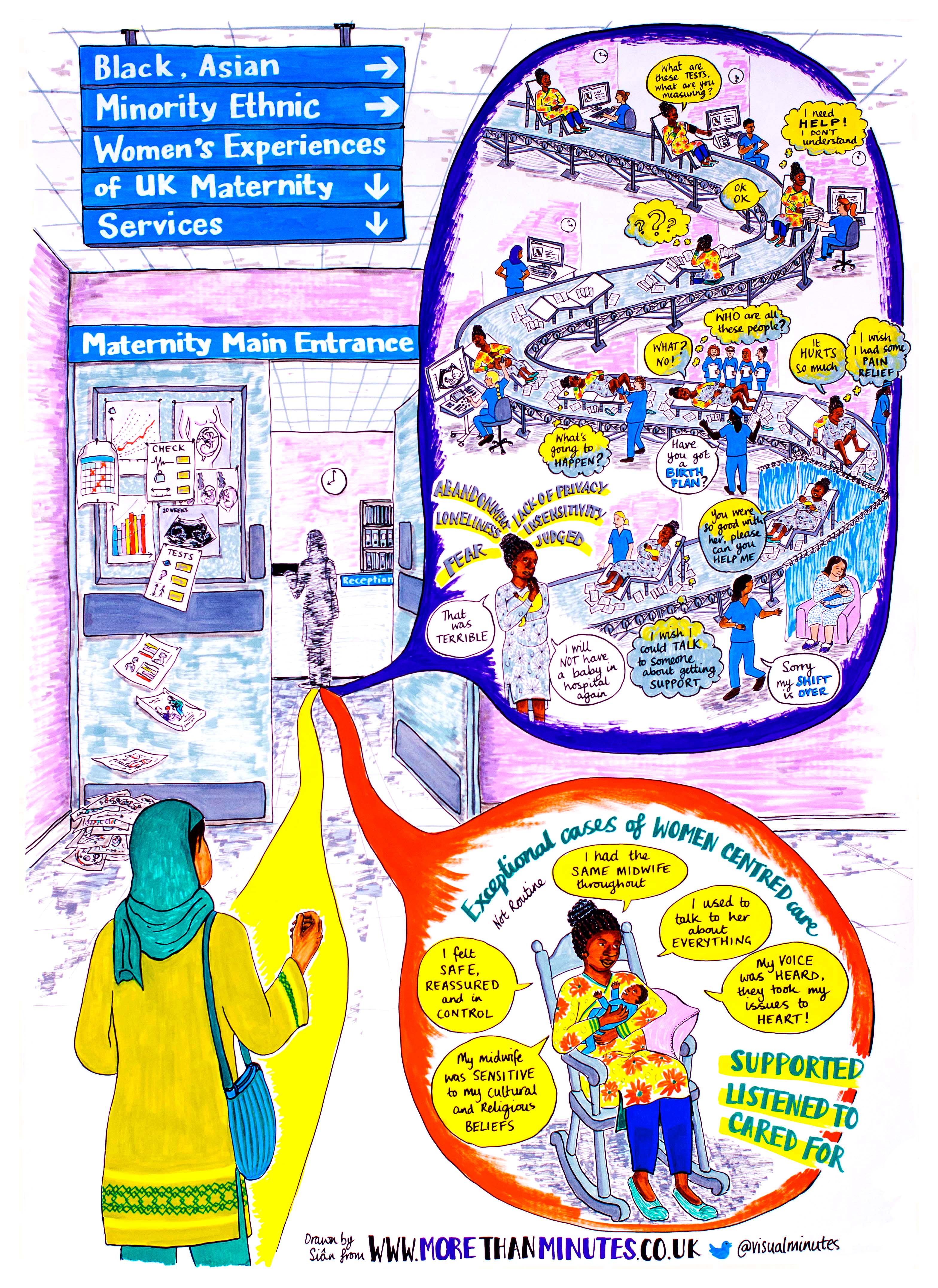

Supplement: Supplementary file 2 — FigureS 1 [file JAN-78-2175-s001.jpg]
